# The associations between calorie tracking, body image dissatisfaction, eating disorders, and menstrual cycle characteristics in resistance-trained athletes

**DOI:** 10.1080/15502783.2024.2433743

**Published:** 2024-11-23

**Authors:** Kimberly SantaBarbara, Eric Helms, Nigel Harris

**Affiliations:** a Auckland University of Technology, Sport Performance Research Institute New Zealand (SPRINZ), Auckland, New Zealand; b California State Polytechnic University, Department of Kinesiology and Health Promotion, Pomona, CA, USA; c Florida Atlantic University, Department of Exercise Science and Health Promotion, Muscle Physiology Laboratory, Boca Raton, FL, USA; d Auckland University of Technology, Human Potential Centre, Auckland, New Zealand

**Keywords:** Strength athlete, menstrual cycle, body image, calorie tracking

## Abstract

**Background:**

While body image dissatisfaction (BID) and eating disorders (EDs) are relatively common in athletes (ranging from 11% to 67% of athletes, depending on the sport) [1], they are also prevalent in weight-class restricted sports (a common format in strength sports), and among physique athletes [2]. These athletes manipulate their nutrition to reach aesthetic or body weight standards and, in that process, may undergo prolonged periods of low energy availability. Low energy availability, defined as consuming insufficient energy for one’s lean mass and exercise activity, can lead to Relative Energy Deficiency in sport (REDs), a syndrome that can impact menstrual cycle (MC) symptoms (and many other aspects of physiology and psychology) [3]. There has not been an investigation into the relationships between these resistance-trained (RT) athletes’ nutritional habits, MC-related symptoms, BIDs, and EDs.

**Methods:**

A survey was implemented to explore the dieting habits, MC characteristics, BID, and EDs in RT females.

**Results:**

64.6% (*n* = 469) of participants reported tracking calories, with a slightly higher percentage of competitive athletes tracking calories 71.8% (*n* = 181) than recreational-level athletes. Competitive athletes were significantly more likely to track calories than recreational-level athletes (*p* = 0.003). When asked what the primary purpose of calorie restriction was, most participants selected weight loss for aesthetic purposes 58.8% (*n* = 356). Competitive athletes were less likely to select weight loss for aesthetic purposes 35.7% (*n* = 77), but weight loss for the purpose of a weight class-based sport was higher at 43.5% (*n* = 94). There were no significant associations between BID and MC characteristics or most MC symptoms and limited associations between EDs and MC characteristics and symptoms.

**Conclusion:**

RT athletes exhibited a higher prevalence of calorie tracking than the general population. Competitive RT athletes were less likely to calorie restrict for aesthetic purposes than non-athletes, but more likely to calorie restrict for the purpose of weight-class-based sports. There were limited significant associations between BID and MC characteristics or MC symptoms, as well as between EDs and MC characteristics. However, there was a significant association between amenorrhea and EDs, which aligns with previous research in this area. Both BID and EDs were significantly associated with MC-based mental health effects; this is likely due to the interconnected nature of mental health concerns, such as EDs with depression and anxiety.

## 1. Introduction

Symptoms related to the menstrual cycle (MC) can impact performance in athletes [1]; however, most research is not on resistance-trained (RT) athletes. RT athletes engage in training that targets muscular strength development, specifically for sports including Olympic weightlifting, powerlifting, Crossfit, throwing sports, and physique sports. RT athletes’ training differs considerably from cardiovascular endurance-based training; therefore, this class of athletes warrants sequestered research to isolate the specific nuances related to RT sports, such as weight-class restriction impacts and sport-related recovery. Further, while body image dissatisfaction (BID) and eating disorders (EDs) are relatively common in all athletes (ranging from 11% to 67% of athletes sampled, depending on the sport and method of assessment) [2]. EDs are also prevalent in weight-class restricted sports (a common format in strength sports) and especially among physique athletes [3]. The prevalence of EDs among female physique athletes is currently unclear, but a recent survey found that 37% of participants were at risk of developing an ED [4]. RT athletes in weight-class restricted sports manipulate their nutrition to reach aesthetic or body weight standards, and in that process, they may undergo prolonged periods of low energy availability [3]. Low energy availability, defined as consuming insufficient energy for one’s lean mass and exercise activity, can lead to relative energy deficiency in sport (REDs), a syndrome which can impact MC symptoms (and many other aspects of physiology and psychology) [5]. Moreover, there has not been a prior investigation into the relationships between these RT athletes’ nutritional habits, MC-related symptoms, BIDs, and EDs.

The MC affects sports training, participation, and performance including limiting training participation and perceived physical capacity [6–8]. For most, the MC causes various symptoms such as pain, and fatigue which for some, can affect athletic performance measures and outcomes [7,9–11]. MC symptoms that affect performance include more than physical symptoms; most female athletes also experience mental health symptoms and changes in their perception of performance, which likely impact their actual performance outcomes [1,12–15].

BID in athletes may stem from the internal pressure that athletes put upon themselves to look a certain way for their sport [16] and from the external pressure coaches put upon athletes, especially if the nature of the sport itself involves aesthetic components or weight-class based categories [17,18]. Athletes in aesthetic- and weight-class sports are more likely to experience MC disturbances than population-matched controls [19,19]. This is likely due to a high prevalence of BID in aesthetic-based, leanness-based, and weight-class based sports, which is extremely common for RT athletes [16,20,21]. Previous research has shown connections between BID and the natural phasic changes of the MC [22], further establishing the link between the MC and BID.

One can experience low energy availability from disordered eating patterns well before they are diagnosed with an ED, especially in exercising females [23–27]. EDs are seen in athletes of mixed ages, sport types, and competitive levels [20,28,29]. EDs combined with low energy availability can alter MC hormonal patterns [30,31], typically manifesting as MC irregularities or amenorrhea [5]. REDs can negatively impact muscular strength and performance and cause more severe health consequences, such as bone mineral density deficiency and suppressed immune function [5]. Nutrition interventions to alleviate REDs, especially in the presence of EDs, contain layers of complexity in how they are delivered. Many interventions presume that athletes track and monitor their nutrition. While nutrition tracking in populations without a predisposition or history of EDs or disordered eating may not risk their development [32], those with a current ED, or a history of EDs, may be negatively impacted by dietary tracking due to the ability to fixate on calorie tracking in an app environment that may appear rewarding, therefore reinforcing restrictive behaviors [33]. Establishing the normative data on dietary practices of RT athletes is necessary to understand if there are links between caloric restriction and dietary tracking leading to REDs in this population.

Most research to date in the area of EDs and REDs does not focus on females who participate in resistance training-based sports, and instead focuses on mixed sport disciplines, or endurance-based athletes [20,29,34–36]. Since resistance training is an important and unique type of exercise that challenges the body differently than cardio endurance exercise training, it is necessary to establish normative data specifically for RT athletes.

General BID and calorie restriction practices that include rapid and significant weight loss (like those commonly used in weight-class-based resistance training sports) can produce negative mental health consequences in athletes, which can lead to EDs [21,36]. EDs can have serious physical health consequences that can lead to a variety of conditions, including REDs, hypothalamic dysfunction, and MC irregularity [36]. Therefore, more knowledge on calorie restriction practices and the connection between BID and EDs with the MC in the RT population is needed. This exploratory survey was implemented to answer the following research questions: What are the calorie tracking and calorie restricting habits of RT females? What is the association between self-reported BID and MC characteristics among RT females? What is the association between ED diagnosis and MC characteristics among RT females? Are there differences in calorie tracking habits, BID, EDs, and MC characteristics between competitive-level RT athletes and recreational-level RT athletes? As this was an exploratory analysis, we did not formulate any hypotheses.

## 2. Methods

An anonymous online survey was implemented to explore the dieting habits, MC characteristics, BID, and EDs in RT females. The survey was designed based on pilot consultations with the target population. The survey was hosted on the Qualtrics Platform (Qualtrics Ltd., Provo, Utah, USA, March 2021). Participants were recruited via social media promotions on the authors’ Instagram pages. The online recruitment poster contained a link that gave interested viewers more information on the data collection procedures. The survey began with an information page that explained the survey’s purpose, objectives, potential risks, and benefits. Participants gave their consent to participate by clicking a “continue” button and commencing the survey. The survey took approximately 10 min to complete and was made available from April 2021 until June 2021. This research was approved by Auckland University of Technology Ethics Committee on 5 March 2021 (AUTEC reference number 21/22).

### 2.1. Procedures

Participants completed the anonymous survey comprising 79 questions, including an optional nutrition and ED section comprising 13 questions. Survey questions were delineated into three sections: 1) the MC characteristics section, 2) the perception of MC effects section, and 3) the nutrition and ED section. The MC characteristics section included questions about period regularity and MC symptoms, timing, and severity. The perception of MC effects section included questions about perceived positive or negative effects on performance, their timing, and severity. The nutrition habits and ED section included questions on calorie tracking habits, calorie restricting habits, ED diagnoses, and BID.

### 2.2. Subjects

Participant inclusion criteria were females aged 18–50 who had not experienced menopause, who participated in full-body resistance training, trained at least twice weekly, and had at least a two-year training history. These qualifiers meant that most participants in this survey had a high level of resistance training experience. Participants who considered themselves competitive-level athletes were asked to self-identify as such, the criteria being that they actively competed in an RT-based sport, which included Olympic Weightlifting, powerlifting, CrossFit, throwing sports, physique-based competitions, and other related strength-based sports, regardless of their level of competition or experience. Those who self-identified as competitive-level were separated for certain analysis purposes and were labeled as competitive athletes and compared to the other participants who were labeled as recreational athletes.

### 2.3. Statistical analysis

Descriptive data, including means, frequencies, standard deviations (SD), percentages, and ranges, were reported for each section to establish normative data in this population. Chi-square tests were used for group comparisons, specifically for analyzing differences in competitive athletes compared to recreational athletes for calorie tracking, BID, EDs, and MC characteristics for categorical data. Chi-square tests were also implemented to explore the relationships between BID and EDs with MC characteristics. Effect sizes were calculated with Cramer’s V. Effect sizes which were reported with degree of freedom of 1 are qualitatively described as follows: ≥0.1 represents a small effect, ≥0.3 represents a medium effect and ≥0.5 represents a large effect. For effect sizes with a degree of freedom of 2: ≥0.07 represents a small effect, ≥0.21 represents a medium effect and ≥0.35 represents a large effect. Finally, for effect sizes with a degree of freedom of 3: ≥0.06 represents a small effect, ≥0.17 represents a medium effect and ≥0.29 represents a large effect [37]. Incomplete or missing data were excluded from the final analysis. The data set followed a normal distribution. Significance was set at the *p* ≤ 0.05 level. All statistical analyses for this research were performed using JASP Team (Amsterdam, The Netherlands, 2023). JASP (Version 0.17) (https://jasp-stats.org/) computer software for Mac.

## 3. Results

Nine hundred and six participants accessed the online survey. Participants who did not meet the survey criteria were removed, which left 809 responses that were analyzed. Participants ranged in age between 18 and 48 years, with a mean age of 27 ± 5.95 years and most on average had been doing resistance training for 6–10 years (30.5%). Most participants spent 5–6 h per week resistance training (44.5%). Two hundred and sixty-seven participants (33%) self-identified as competitive-level athletes, with most competing at the regional or national level (88.3%). Further information on competitive athletes is shown in Table 1.

**Table 1.** Participant demographic information.

	Percent	Count
**Survey question: What sport do you currently compete in?**		
Weightlifting	11.1%	32
Powerlifting	51.7%	149
Strongman/Strongwoman	2.1%	6
Physique based sport	11.1%	32
CrossFit	22.8%	31
Other	12.9%	37
**Q: What is the highest level you have competed in your sport?**		
Regionally/Locally	48.8%	141
State or National Level	36.7%	106
International or elite level	14.6%	42
**Q: How many years have you competed in this sport?**		
1–2 years	44.4%	127
3–4 years	32.5%	93
5–6 years	9.8%	28
7 or more years	11.5%	33
**Q: How many hours a week do you participate in resistance or strength training?** 3–4 hours per week	105	24.5
5–6 hours per week	207	41.65
7 or more hours per week	168	33.8

(This table has been adapted from a previous publication, SantaBarbara, et al., (2024)).

### 3.1. Calorie tracking and calorie restricting habits

A 64.6% (*n* = 469) of participants reported tracking calories, with a slightly higher percentage of competitive athletes tracking calories 71.8% (*n* = 181) than recreational athletes. Competitive athletes were significantly more likely to track calories than recreational athletes (*p* = 0.003) with a small effect size (0.111). A 81.3% (*n* = 617) of participants said they had purposefully dieted or restricted calories in the past 2 years. Competitive athletes gave a similar response of 83.7% (*n* = 220). When asked what the primary purpose of calorie restriction was, most participants selected weight loss for aesthetic purposes 58.8% (*n* = 356). Competitive athletes were less likely to select weight loss for aesthetic purposes 35.7% (*n* = 77), but weight loss for the purpose of a weight class-based sport was higher at 43.5% (*n* = 94). Most participants said they calorie restricted once a year or less, 43% (*n* = 260). There was no significant difference between competitive athletes and recreational athletes in their frequency of calorie restriction (*p* = 0.153). See Table 2 for further information on calorie restriction and dieting habits.

**Table 2.** Nutritional habits breakdown.

	Non-Athletes		Strength Athletes	
	Percent	Count	Percent	Count
**What is the purpose of your calorie restriction practices?**				
Weight loss for aesthetic reasons	58.8%	356	35.7%	77
Weight loss for weight class-based sport	18.2%	110	43.5%	94
Weight loss for general health	11.6%	70	6.5%	14
Weight loss for improved sport performance	7.3%	44	11.6%	25
Other	4.1%	25	2.8%	6
**How often do you attempt caloric restriction in a given year?**				
Once a year or less	43.0%	260	41.7%	90
2–3 times a year	39.7%	240	44.4%	96
4–5 times a year	9.9%	60	6.5%	14
Other	9.5%	45	7.5%	16

### 3.2. BID and EDs

BID prevalence was captured with the question, “How often are you dissatisfied by your weight/shape?” with the following answer choices: “Not at all,” “On occasion,” “Often,” “Almost all the time.” Participants answered “Almost all the time” 18% (*n* = 134), “Often” 25.4% (*n* = 189), “On occasion” 43.2% (*n* = 321), and “Not at all” 13.4% (*n* = 100) of the time. Competitive athletes were significantly less likely to answer, “Almost all the time” (*p* = 0.003) than recreational athletes with a medium effect size (0.14). ED prevalence was captured with the question, “In the past two years have you been diagnosed with an eating disorder?” most participants answered “no,” 95% (*n* = 700). Competitive athletes similarly selected “no” 95.6% (*n* = 248) of the time. All participants were then asked if they had any concerns that they may be developing an ED, to which 20.5% (140) answered yes.

### 3.3. The connection between BID and EDs with MC characteristics

There were no significant associations between BID and MC characteristics or most MC symptoms; see Table 3 for further information. The presence of an ED and the MC characteristic amenorrhea were significantly associated (*p* = 0.01) with a medium effect size (0.293). Other MC characteristics and most other MC symptoms had no significant association with EDs. See Table 3 for further information and Chi-Square test results.

**Table 3.** Chi-square test results.

Questions	X^2^	df	*p*	Effect Size
BID and strength athletes versus non athletes	16.4	4	.003	.140
BID and MC based mental health effects	21.6	4	.001	.179
BID and periods regular	9.0	6	.173	.080
BID and digestive issues	26.1	4	.001	.196
BID and MC length	6.0	4	.198	.082
BID and amenorrhea	4.7	4	.320	.169
BID and MC has negative impact on training	13.3	4	.010	.140
BID and cramps	4.9	3	.182	.081
EDs and strength athletes	2.9	1	.086	.063
EDs and amenorrhea	13.6	2	.001	.293
EDs and MC has negative impact on training	.6	2	.755	.029
EDs and MC based mental health effects	5.8	1	.016	.089
EDs and digestive distress	3.7	2	.155	.076
EDs and periods regular	1.5	4	.832	.033
EDs and MC length	.185	2	.912	.016
EDs and digestive distress	.9	1	.336	.035
EDs and cramps	.08	1	.928	.003
Strength athletes versus non-athletes calorie track	8.9	1	.003	.111
Strength athletes and caloric restriction frequency	6.7	4	.153	.108
Purpose of calorie restriction and periods regular	5.3	2	.071	.087
Frequency of calorie restriction and periods regular	14.6	8	.067	.114
Frequency of calorie restriction and MC has negative impact on training	10.4	8	.236	.096

## 4. Discussion

The research aims of this survey were to establish the calorie tracking and restricting habits of RT athletes and to establish the relationship between BID and EDs with MC characteristics in this population. Further, a secondary aim was to compare the habits of competitive RT athletes with recreational-level RT athletes. Our principal findings were that calorie tracking may be more prevalent in RT athletes than in the general population [38]. Further, there were few significant associations between BID, EDs, and MC characteristics. Finally, competitive-level RT athletes answered similarly to recreational-level RT athletes in many questions, with some differences in calorie tracking prevalence, reasoning, and BID frequency and severity.

About 65% of participants tracked their calorie intake regularly, with competitive athletes significantly more likely to track calories. This is notably higher than previous research on the general population, which showed that 12.3% of young adult females [38] and 26% of female university students [39] tracked calorie intake using an app. Previous qualitative research has shown that calorie tracking can be beneficial in some contexts but also has the capacity to become an obsessive habit [40]. Although calorie tracking by itself may not be a cause for concern in this population, this habit has been associated with heightened ED severity and disordered eating behavior in the general population [38,41–43]. Further research is needed to establish the connections between calorie tracking and ED pathology in RT athletes.

Most respondents stated they restricted calories at least once in the past 2 years (81.3%), which is similar to previous research on aesthetic-based athletes, such as figure skaters and weight-class-based athletes, who regularly engage in calorie-restriction [2,44]. Most calorie restriction in the present survey was for weight loss for aesthetic reasons (58.8%), even when physique athletes were removed (as it could be argued that for physique competitors, aesthetic reasons are also for the purpose of sport performance). The general population may feel societal pressure to meet specific appearance-based goals, which for many includes weight loss [17,45]; this may be a similar sentiment that RT females also experience, as was shown in the present survey results. Competitive athletes in the present survey, on the other hand, differed in their reason for calorie restriction; fewer cited aesthetic reasons (35.7%) for caloric restriction, and more cited competition in a weight class-based strength sport (43.5%). This finding demonstrates that RT athletes may differ from athletes of mixed sports, as RT athletes may be more concerned, on average, about weight-based shape or appearance than non-aesthetic-based athletes [45–47]. Thus, further research is needed to determine why and to what degree RT athletes may differ from other types of athletes, as this may indicate a higher risk for EDs among RT athletes.

In our survey, 43.4% of participants reported experiencing BID “Often” or “All the time,” which is lower than some research on mixed sports athletes, where 50–83% of surveyed athletes reported BID [46]. This difference in BID levels measured in previous research could be due to different question phrasing, or may be representative of differences in sport type, age, competitive level, or the cultural background of the participants surveyed. Competitive athletes in the present survey were significantly less likely to experience BID “Often” or “All the time” than recreational athletes. This finding contrasts with previous research that shows athletes at higher competitive levels, and athletes in general, have a higher likelihood of BID than the general public [48,49]. However, not all research agrees, as some data indicate lower levels of BID among athletes than non-athletes [49]. These contrasting findings may indicate that BID differs by sport type and level of competition.

In this survey, a low level of participants (5%) had been diagnosed with an ED, which is lower than previous research on the prevalence of EDs in other athletes, which has ranged from 18% to 42% [17,47,50]. Differences could be due to different question phrasing or, once again, the athlete population, including sport type, age, competitive level, and cultural background. The question used in the present survey to establish EDs asked specifically about ED diagnoses. However, much of the previous research asks questions related to ED symptoms, indicating that phrasing can have a substantial impact on findings. Further, not all individuals with disordered eating patterns necessarily seek medical help which could result in a diagnosis. Competitive athletes in this survey had a similar prevalence of ED diagnoses as recreational athletes, which also matches rates reported in a literature review by Chapa et al. (2022).

This research demonstrated that BID measures had no significant relationship with MC characteristics, including cycle length and regularity. This aligns with previous research on college-level athletes [49] but differs from other research where a connection between BID and MC phase in the general population was established [22]. Based on these conflicting findings, further research is warranted to better elucidate the connections between BID and the MC in different populations.

ED diagnosis was significantly associated with amenorrhea, which aligns with previous research on REDs and the Female Athlete Triad [5,23] and fits with the current model of understanding in low energy availability and MC hormone regularity. Further, BID was associated with a negative perception of the MC’s effects on training, but ED diagnosis was not. This is a novel area of exploration of this population, and further research is needed to establish associations between ED and BID with perceptions of MC effects. Both high BID and the presence of EDs were significantly associated with MC symptom-based mental health concerns. Moreso, the most frequently cited mental health issues connected to the MC were anxiety and depression, which is supported by previous research linking mental health conditions such as anxiety and depression with BID and ED [20,48–50]. Mental health and its connection with the MC are essential areas of research that warrant further investigation, especially concerning BID and EDs in the RT population, which to date is relatively understudied.

## 5. Limitations

This survey has an inherent memory recall bias that may limit the accuracy of our findings. Since the focus of this survey was to explore the relationship between BID and EDs with the MC, rather than to investigate in-depth levels of BID or signs of EDs, the conclusions surrounding BID or ED prevalence in this population are also limited. To keep this survey at a reasonable length and focused on MC characteristics and other training-based data, we did not use longer form, validated measures for BID. Similarly, the primary question utilized for EDs was a question which asked if participants had received an ED diagnosis without other questions about symptomology as, again, this was not the primary focus of this survey and length was a concern. Specific ED classifications were also not isolated. Therefore, all ED diagnoses were analyzed as one data point in this survey, which is a limitation, as some EDs may not be related to amenorrhea, which potentially influenced our findings. This data should be viewed as exploratory data for BID and EDs in this population.

## 6. Conclusion

RT females exhibited a higher prevalence of calorie tracking than the general population. Competitive-level RT athletes were less likely to calorie restrict for aesthetic purposes than recreational athletes, but more likely to calorie restrict for the purpose of weight-class-based sports. There were limited significant associations between BID and MC characteristics or MC symptoms as well as between EDs and MC characteristics. However, there was a significant association between amenorrhea and EDs, which aligns with previous research in this area. Both BID and EDs were significantly associated with MC-based mental health effects; this is likely due to the interconnected nature of mental health concerns like depression and anxiety. Future research that uses validated questions to establish BID and ED prevalence is needed to expand on this survey. Moreso, active MC hormonal monitoring in RT females would be an important step to move beyond the memory recall limitations of survey data. Such monitoring studies should have a focus on mental health effects, which would allow clearer relationships between BID, ED, and MC effects in the RT population to be established.

## Data Availability

The full data set from this survey can be made available by request to the corresponding author.
